# Temperature-Dependent Phase Behaviors in Cylinder-Forming Block Copolymers

**DOI:** 10.3390/ijms10052169

**Published:** 2009-05-15

**Authors:** Dae Up Ahn, Erol Sancaktar

**Affiliations:** 1 Department of Mechanical Engineering, University of Colorado, Boulder, CO 80309-0427, USA; 2 Department of Polymer Engineering, The University of Akron, Akron, OH 44325-0301, USA

**Keywords:** block copolymers, block copolymer self-assembly, dynamic mechanical properties, orientation transition of block copolymer nano-cylinders, order-disorder transition of block copolymers

## Abstract

We demonstrate that the temperature-dependent phase behaviors of parallel and perpendicular cylinder-forming block copolymers are governed by domain-domain segregation forces inherently present in block copolymer material itself. With increasing temperature, a parallel cylinder-forming block copolymer experienced a parallel cylinder straightening process before the order-disorder transition (ODT) and did not show long-range composition fluctuations near the ODT temperature due to the weak segregation forces between the block domains. A perpendicular cylinder-forming block copolymer with a strong segregation force between the block domains displayed cylinder orientation transition from perpendicular to parallel below the ODT temperature. On the other hand, a perpendicular cylinder-forming block copolymer material with an exceptionally strong segregation force between the block domains maintained its initial perpendicular cylinder orientation up to near the ODT temperature. In both cases of perpendicular cylinder-forming block copolymers, submicrometer-scale long-range composition fluctuations were observed well above the ODT temperature due to their intrinsically strong segregation forces between the block domains.

## 1. Introduction

The majority of studies on temperature-dependent phase behaviors of block copolymers have concentrated on the phase behaviors of weakly ordered block copolymers near the lamellar-to-disorder, cylinder-to-disorder, cylinder-to-sphere or sphere-to-disorder transitions (i.e., in the relatively high temperature region) [1–5], even though information on phase behaviors of strongly ordered block copolymers is required for nanotechnological applications of block copolymers. In the temperature region lower than the order-disorder transition (ODT), however, lamellar and cylinder microphases exhibit additional complexities in their temperature-dependent phase behaviors due to the presence of anisotropic phases arising from the preferential microdomain orientations either parallel or perpendicular to the surface, which also largely depend on the temperature. In order to understand and/or minimize such complexities, researchers have modified the microphase orientation macroscopically by applying flow fields before their experiments on temperature-dependent phase behaviors of lamellar and cylinder microphases [6–9]. However, it is not clear whether the microphase structures of flow-induced block copolymer materials, observed at a sufficiently high temperature above the glass transition temperatures of block components, are originating from the intrinsic phase behavior of initially stable microstructure or the temporary phase transition of kinetically frozen microstructures.

According to our recent experimental results [10,11], thermodynamically stable block copolymer cylinders oriented either parallel or perpendicular to the surface can be achieved over the whole sample area and thickness without any applications of external fields, neutral surfaces, and solvent annealing [12,13]. We showed that the intrinsic preference for parallel or perpendicular cylinder orientation of a cylinder-forming block copolymer was readily controlled by adjusting the molecular weight of homopolymer blended. Parallel cylinder orientation was obtained on an energetically preferential substrate when the molecular weight ratio between the minority block component and its corresponding homopolymer (i.e., higher molecular weight PS component/lower molecular weight PS component), *r* was larger than 2. In the molecular weight ratio between 1 and 2, on the other hand, overall perpendicular cylinder orientation was spontaneously produced even on an energetically preferential substrate when the thickness of miscible samples was smaller than ~ 10 μm. Such parallel-to-perpendicular cylinder orientation transition originated from the strong intermolecular interaction between the minority block component and its homopolymer blended, which significantly enhanced the enthalpic preference for the perpendicular cylinder orientation (as well as the incompatibility between the block components) to overcome the parallel cylinder-inducing interfacial interaction between the substrate and its preferential block component. In this study, by using stable block copolymer cylinders oriented either parallel or perpendicular to the surface, we demonstrate that the temperature-dependent phase behaviors of cylinder-forming block copolymers depend on their preference for parallel or perpendicular cylinder orientation, whose strength is directly proportional to the strength of segregation force (i.e., incompatibility) between the block domains.

## 2. Experiments

A commercial grade of parallel cylinder-forming polystyrene-*block*-polyisoprene-*block*-polystyrene (SIS, Vector 4113, DEXCO Polymers Co.) triblock copolymer was used as received. This triblock copolymer is highly asymmetric in block composition, which contains 15.4 wt.% of polystyrene (PS). Four different PS’s directing a spontaneous parallel or perpendicular cylinder orientation, when they were incorporated with the SIS, were synthesized by anionic polymerization using *sec*-butyllithium as initiator, benzene as solvent, and standard high-vacuum techniques: 1) PS2k (the weight average molecular weight, *M*_w_ = 2.0 kg/mol with *M*_w_/*M*_n_ = 1.10 by gel permeation chromatography, GPC); 2) PS4k (*M*_w_ = 4.0 kg/mol with *M*_w_/*M*_n_ = 1.09, by GPC); 3) PS12k (*M*_w_ = 12.2 kg/mol with *M*_w_/*M*_n_ = 1.08, by GPC); and 4) PS15k (*M*_w_ = 15.4 kg/mol with *M*_w_/*M*_n_ = 1.03, by GPC).

The block copolymer and its homopolymer mixture samples were prepared by first dissolving a predetermined amount of SIS triblock copolymer and its PS homopolymer in toluene (3.5 wt. % in solution) with 0.05 wt.% of antioxidant (Irganox 1010, Ciba-Geigy Group). The solution was cast onto the Teflon substrate, and the solvent was slowly evaporated under ambient conditions for two days. Finally, all the samples were annealed in a vacuum oven at different annealing temperatures, which were well above the glass transition temperatures of PS block and PS homopolymers. Five different ~10 μm-thick SIS triblock copolymer and its PS homopolymer mixture films were prepared: 1) Neat SIS; 2) SIS + PS2k (90 wt.% of neat SIS triblock copolymer and 10 wt.% of PS2k); 3) SIS + PS4k (90 wt.% of neat SIS triblock copolymer and 10 wt.% of PS4k); 4) SIS + PS12k (90 wt.% of neat SIS triblock copolymer and 10 wt.% of PS12k); and 5) SIS + PS15k (90 wt.% of neat SIS triblock copolymer and 10 wt.% of PS15k).

The temperature-dependent block copolymer morphologies were identified with a Nanoscope III from Digital Instruments. The AFM was operated in the tapping mode under ambient conditions using commercial silicon microcantilever probe tips (Veeco, RTESP) with spring constants ranging from 20 to 80 N/m as specified by the manufacturer. Thermal histories of the block copolymer samples used in AFM measurements were controlled so that they would be similar to those experienced in dynamic mechanical measurements, as summarized in Table 1. We note that the AFM-determined cylinder orientation of a block copolymer used represented its characteristic orientation throughout the whole sample area and thickness because the surface AFM image of the block copolymer sample was identical to its depth AFM image as long as the sample thickness was smaller than ~ 10 μm [10,11].

The dynamic mechanical properties of the SIS triblock copolymer and its mixtures were measured with an Advanced Rheometric Expansion System (ARES, Rheometric Scientific). The measurements were conducted in shear oscillatory mode using 25 mm-diameter parallel disk geometry with gap setting of ~ 2 mm. In order to prepare a ~ 2 mm-thick block copolymer sample for dynamic mechanical measurements, ~ 10 μm-thick film samples solution-cast onto Teflon substrate were stacked layer-by-layer, and then they were gently pressed at ~ 100 °C to minimize the effects of interfaces between the films on the dynamic mechanical properties of block copolymers used as much as possible. Thus, the overall cylinder orientation formed in the ~ 10 μm-thick block copolymer sample is expected to be preserved in the ~ 2 mm-thick disk sample as well. The block copolymer samples were annealed at a selected temperature for 10 min before starting dynamic mechanical measurements, and all data were collected for ~ 20 min per each selected temperature under a nitrogen atmosphere to prevent thermal degradation of the samples. The range of angular frequency (*ω*) was from 0.05 to 100 rad/s and the range of temperature was selected from 110 °C to 250 °C, depending on the sample. The strain amplitude of 2% was selected to be large enough for accurate torque signals and small enough to keep the material response in the linear region, as well as minimizing any shear effect on microphase phase separation processes of block copolymer mixtures.

## 3. Results and Discussion

### 3.1. Phase Behaviors of a Highly Asymmetric Parallel Cylinder-Forming Block Copolymer

**Figure 1.**
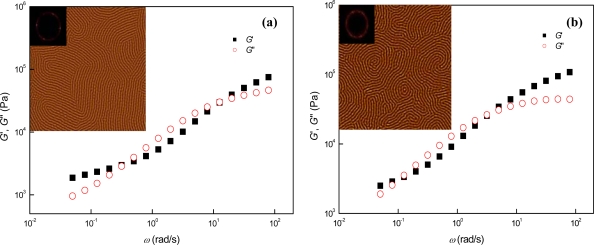

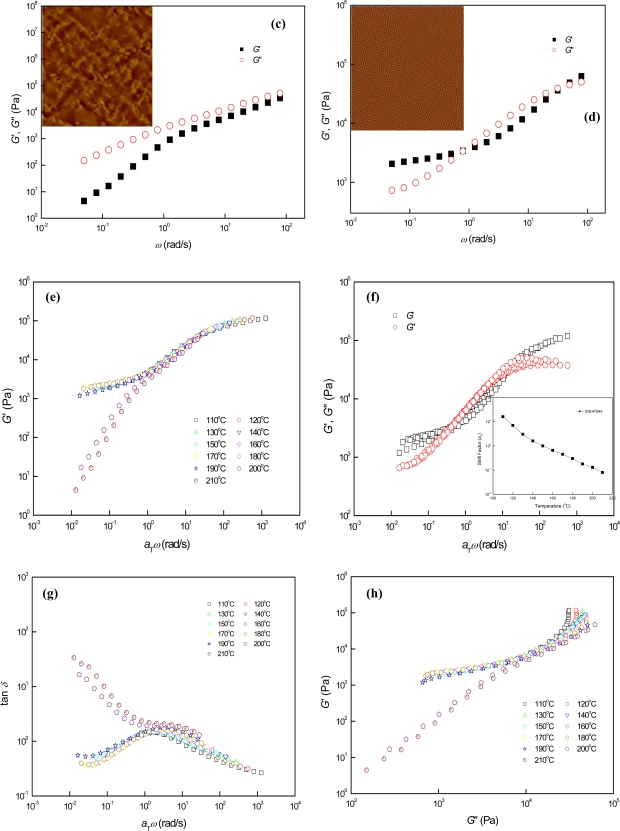
AFM images and dynamic mechanical properties of neat SIS at different temperatures: (a) SIS, (b) SIS150, (c) SIS170, (d) SIS210, (e) logarithmic TTS plot of *G*′, (f) logarithmic TTS plots of *G*′ and *G*″, (g) logarithmic TTS plot of tan *δ*, and (h) logarithmic plot of *G*′ vs. *G*″. The inset of Figure 1 (f) is shift factors (*a*_T_) used (reference temperature, *T*_r_ = 150 °C). The size of AFM images is 2 × 2 μm^2^.

Figure 1 shows the phase behaviors of parallel cylinder-forming SIS triblock copolymer at different temperatures. The AFM images were obtained from ~10 μm-thick samples annealed on the Teflon substrates, and the inset of the AFM image is a 2D fast Fourier transform (FFT) of the image. In the temperature range from 130 to 150 °C [Figures 1(a) and (b)], the straightness of parallel cylinders was enhanced with increasing temperature due to temperature-proportional increase of preference for reducing the interfacial area between the block domains. The 2D FFT’s included as insets in the AFM images of Figure 1 clearly demonstrate that the directional order of parallel cylinders in the *x*,*y*-plane is enhanced with increasing temperature. At 170 °C [Figure 1 (c)], a spherical microphase containing small amounts of parallel cylinders was identified due to the volume fraction of PS block being smaller than the critical volume fraction for cylindrical microphase [14]. Following the order-order transition (OOT) from parallel cylinders to spheres, the PS spheres were gradually coagulated and disordered (i.e., lattice disordering) with increasing the temperature, and finally an overall melt state (i.e., ODT) was identified at 210 °C as shown in Figure 1(d). Similar temperature-dependent phase behaviors were also identified from the dynamic mechanical measurements on the SIS triblock copolymer. Because the value of elastic shear modulus (*G*′) at the *ω* of 0.05 rad/s increased from 1,870 to 2,050 Pa as the temperature increased from 150 to 170 °C [Figures 1(b) and (c)], we can expect a phase transition from a relatively low elastic microphase (in this case, cylindrical microphase) to a further elastic microphase (in this case, spherical microphase) during the temperature range. It is also notable that the rubbery plateau-like region (whose range is mainly affected by the number of physical crosslinks) gradually spanned to low *ω* region in spite of the increase of temperature [Figures 1(b) and (c)]. Thus, the temperature-independent abnormal enhancement of elasticity can be attributed to the increased number of physical crosslinks (i.e., the increased number of PS domains) after the microphase transition of SIS triblock copolymer from parallel cylinders to spheres. After the OOT (i.e., in the temperature region above ~ 170 °C), the values of *G*′ in the low *ω* region gradually decreased and its slope steadily increased with elevating the temperature (i.e., the elasticity of spherical microdomains decreased during the lattice disordering process), and then the slope finally approached to 2 at 210 °C (i.e., a homogeneous melt state at 210 °C) as shown in Figure 1(d).

The OOT and ODT temperatures of a block copolymer melt can also be further elaborately determined by the dynamic mechanical measurements since each of the ordered microphases displays somewhat different viscoelastic behaviors, while the disordered melts exhibit a viscous flow [15–18]. Temperature-reduced plots such as logarithmic plots of time-temperature superposed *G*′ vs. *ω* and logarithmic plots of *G*′ vs. *G*″ (dynamic loss shear modulus) have been exploited to evaluate the OOT and ODT temperatures of a block copolymer [15,16]. In case of a lamella-forming symmetric diblock copolymer, the failure of time-temperature superposition (TTS), occurring as a thermal concentration fluctuation induced breakdown of thermodynamic simplicity, has been assumed to occur at the ODT temperature [15]. On the other hand, in case of sphere-forming asymmetric block copolymers, which experienced the OOT from cylindrical to spherical microstructure before the ODT, the lattice-disordering transition (LDT) of the spherical microdomains was identified before the occurrence of the ODT [16]. Hence, in that case, the ODT temperature was defined as a temperature above which the spherical microdomains disappear completely. Therefore, the onset temperature of TTS failure in the logarithmic *G*′ vs. *G*″ plots was regarded as the LDT temperature, and the further extended failure of TTS above the LDT temperature was considered to represent the process of lattice disordering. In other words, it was assumed that during heating, the initially ordered phase transferred to lattice disordered phase, and then converted to completely disordered phase followed by its short range-ordered thermal fluctuation. We note that the ODT temperature predicted by the former argument was lower than the temperature expected by the latter one, especially when the block copolymer microstructures had extensive lattice ordering in their ordered state. In case of neat SIS, the former argument based on the logarithmic plots of time-temperature superposed *G*′ vs. frequency [Figure 1(e)] predicted the ODT at ~ 190 °C, while the latter argument based on the logarithmic plots of *G*′ vs. *G*″ [Figure 1(h)] predicted the LDT at ~ 190 °C and the ODT at ~ 200 °C. Even though the latter argument appears to be more reliable, conceptually, for determination of the ODT temperature of neat SIS, since it contains large amount of spherical lattice ordering in its ordered state at 170 °C [Figure 1(c)], the LDT and the ODT identified by the logarithmic plots of *G*′ vs. *G*″ do not match precisely with those observed directly by AFM measurements. We note that the neat SIS shows a notable spherical lattice disordering even at 170 °C as shown in Figure 1(c). Thus, the LDT of sphere microphases will occur at a temperature lower than the onset temperature of TTS failure in the logarithmic *G*′ vs. *G*″ plots, and as a consequence, the onset temperature will mainly originate from the global disappearance of spherical lattice ordering, rather than the short-range disordering of spherical lattices. We can also confirm the LDT termination (i.e., the ODT) at ~ 190 °C based on the TTS failure of tan *δ* in the low frequency region of the time-temperature superposed tan *δ* vs. frequency plots. As shown in Figure 1 (g), the thermorheological complexity started to occur at ~ 190 °C, and then a homogenous melt state was observed above ~ 200 °C.

Based on AFM and dynamic mechanical measurements, therefore, we can differentiate the temperature-dependent microphases of the parallel cylinder-containing SIS triblock copolymer into the following five distinctive microphases and their transitions: 1) Parallel cylinder microphase from 130 °C to 150 °C; 2) Phase transition from cylinder to sphere in the temperature range between 150 °C and 160 °C (i.e., the OOT temperature ~ 155 °C); 3) Spherical microphases and their lattice disordering in the temperature range between 160°C and 180 °C (the LDT temperature ~ 170 °C); 4) the LDT termination, i.e., the disappearance of spheres and transient composition fluctuation-driven microphases at 190°C (i.e., the ODT temperature ~190 °C); and 5) Homogeneous melt above ~ 200 °C. It is notable that the *G*′ (*ω*) values at 0.05 rad/s were 2,130, 1,870, 2,100, 2,050 Pa, with 140, 150, 160, 170 °C, respectively. Thus, we estimate that the OOT from parallel cylinder to sphere takes place at ~ 155 °C, and the lattice disordering of the spheres starts to occur at ~ 165 °C. In addition, we note that both the parallel cylinder stretching and the short-ranged LDT processes, identified with AFM measurements in the temperature range from 130 °C to 150 °C and from 160 °C and 180 °C, respectively, are not rheologically detected since the orders of those phase transitions are too high to be observed in the temperature-reduced rheological plots. It is also notable that the shift factors used for the TTS of *G*′ were identically applied to the TTS of *G*″, as shown in Figure 1(f). This identical application of shift factors implies that the short-ranged temperature-dependent phase transitions of neat SIS, such as parallel cylinder stretching and short-ranged LDT processes, which occur in the temperature region lower than ~ 190 °C, should not significantly affect the thermorheological simplicity of neat SIS, and consequently we can effectively use the TTS plots to predict the ODT temperature of neat SIS.

### 3.2. Phase Behaviors of Parallel Cylinder-Forming Block Copolymer Mixtures with a Relatively Weak Segregation Force between the Block Domains

**Figure 2.**
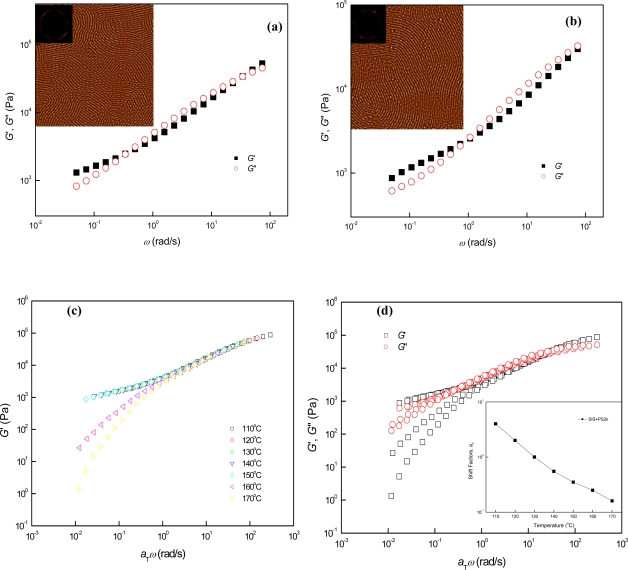

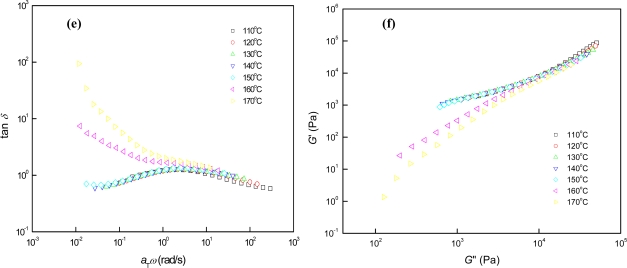
AFM images and dynamic mechanical properties of SIS + PS2k at different temperatures: (a) SIS + PS2k, (b) SIS + PS2k150, (c) logarithmic TTS plot of *G*′, (d) logarithmic TTS plots of *G*′ and *G*″, (e) logarithmic TTS plot of tan *δ*, and (f) logarithmic plot of *G*′ vs. *G*″. The inset of Figure 2 (d) is *a*_T_ used (*T*_r_ = 130 °C). AFM images were obtained from ~10 *μ*m-thick samples annealed on the Teflon substrates, and the size of the images is 2 × 2 μm^2^.

Temperature-dependent microphase behaviors of parallel cylinder-containing SIS + PS2k and SIS + PS4k are simpler than those of neat SIS, because those do not experience the OOT from cylinder to sphere, since their volume fractions of PS component are higher than the critical volume fraction for cylindrical microphase over their experimental temperature range [11]. In the temperature range from 130 °C to 150 °C, the straightness of parallel cylinders in SIS + PS2k was enhanced with increasing temperature, because the temperature-proportional decrease of segregation forces between the block domains reduced the interfacial area between the PS and PI block domains (Figure 2). After the parallel cylinder straightening process, the parallel cylinders in SIS + PS2k abruptly disappeared without any notable composition fluctuations, and homogeneous melt state started to be observed at the relatively low temperature of 170 °C due to the weak segregation force between the domains.

The weak domain-domain incompatibility of SIS + PS2k can also be confirmed by dynamic mechanical measurements. Below the ODT, the values of *G*′ in the low frequency region gradually decreased with increasing temperature without a notable rubbery plateau-like region [Figures 2(a) and (b)]. Additionally, following thermorheologically simple behavior in the 130 °C to 150 °C temperature range, a sudden failure of TTS started to occur at ~160 °C, as shown in Figures 2(c), (d), (e) and (f). Thus, we can classify the temperature-dependent microphases of parallel cylinder-containing SIS+PS2k into the following three distinctive microphases and their transitions: 1) Parallel cylinder straightening in the temperature range from 130 °C to 150 °C; 2) Sudden phase transition from parallel cylinder microphase to homogeneous melt phase at 160 °C (i.e., ODT ~ 160 °C); and 3) Homogeneous melt states above 170 °C. It is notable that the SIS + PS2k does not display long-range (i.e., rheologically measurable) composition fluctuations above the ODT temperature since the segregation force between the block domains is not strong enough to maintain a thermally localized transient inhomogeneous phase near the ODT temperature.

**Figure 3.**
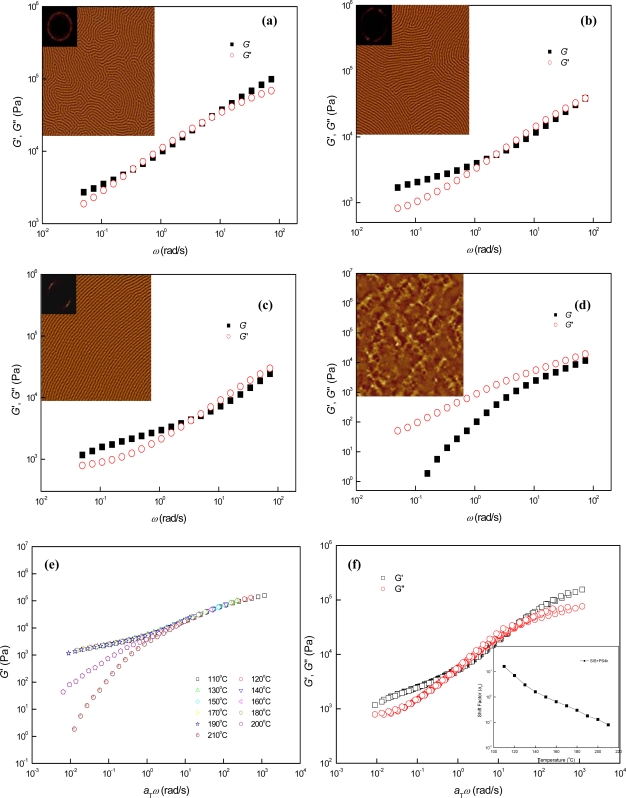

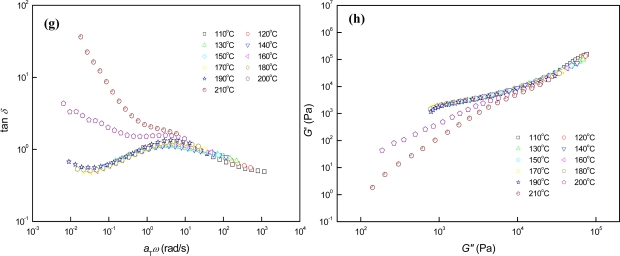
AFM images and dynamic mechanical properties of SIS + PS4k at different temperatures: (a) SIS + PS4k, (b) SIS + PS4k170, (c) SIS + PS4k190, (d) SIS + PS4k210, (e) logarithmic TTS plot of *G*′, (f) logarithmic TTS plots of *G*′ and *G*″, (g) logarithmic TTS plot of tan *δ*, and (h) logarithmic plot of *G*′ vs. *G*″. The inset of Figure 3 (f) is *a*_T_ used (*T*_r_ = 150 °C). AFM images were obtained from ~10 *μ*m-thick samples annealed on the Teflon substrates, and the size of the images is 2 × 2 μm^2^.

Similar temperature-dependent phase behaviors were observed in the parallel cylinder-forming SIS + PS4k, because it also possessed a weak segregation force between block components. In the temperature range from 130 °C to 190 °C, the straightness of parallel cylinders in SIS + PS4k was enhanced with increasing temperature due to the temperature-proportional increase of miscibility between the block domains [Figures 3(a), (b), and (c)]. After the parallel cylinder straightening process, the parallel cylinders in SIS+PS4k abruptly disappeared to homogeneous melt state without any rheologically detectable transient composition fluctuations since the segregation forces (i.e., incompatibility) between the block domains is not strong enough to maintain a thermally localized inhomogeneous phase near the ODT temperature [Figure 3(d)]. As shown in Figures 3(e), (f), (g) and (h), a sudden failure of TTS started at 200 °C after thermorheologically simple behavior from 130 °C to 190 °C, due to the absence of lattice disordering or composition fluctuation before the ODT. Thus, we divide the temperature-dependent microphases of parallel cylinder-forming SIS + PS4k, which possesses a relatively weak segregation force between the block domains, into the following three distinctive microphases and transitions: 1) Parallel cylinder straightening in the temperature range from 130 °C to 190 °C; 2) Sudden phase transition from parallel cylinder microphase to homogeneous melt phase between 190 °C and 200 °C (i.e., ODT ~ 195 °C); and 3) Homogeneous melt states above 200 °C. From the temperature-dependent phase behaviors of SIS + PS2k and SIS + PS4k, we conclude that a parallel cylinder-forming block copolymer material with a relatively weak incompatibility between the block domains did not show long-range composition fluctuations near the ODT temperature due to its weak segregation forces between the block domains. In this weak segregation limit, the ODT of SIS + PS4k was higher than that of SIS + PS2k, since the incompatibility between block domains simply increased by increasing the molecular weight of the blended homopolymer.

### 3.3. Phase Behaviors of Perpendicular Cylinder-Forming Block Copolymer Mixture with a Strong Segregation Force between the Block Domains

**Figure 4.**
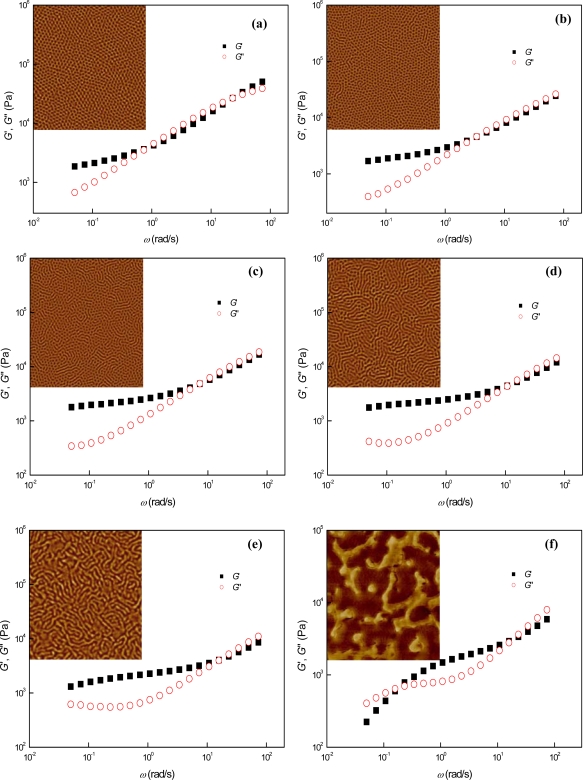

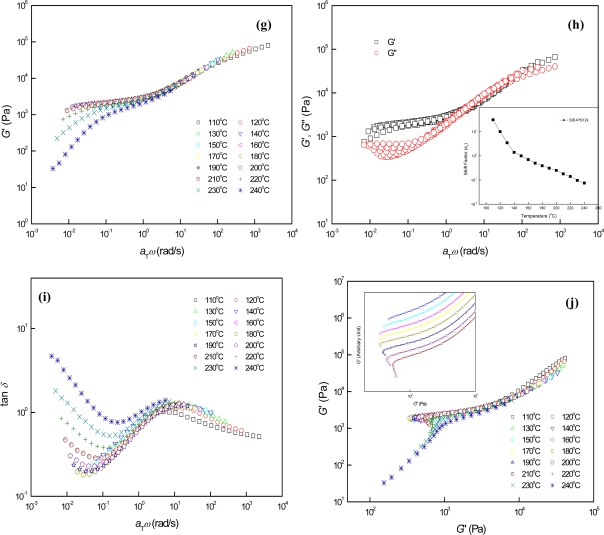
AFM images and dynamic mechanical properties of SIS + PS12k at different temperatures: (a) SIS + PS12k, (b) SIS + PS12k150, (c) SIS + PS12k170, (d) SIS + PS12k190, (e) SIS + PS12k210, (f) SIS + PS12k230, (g) logarithmic TTS plot of *G*′, (h) logarithmic TTS plots of *G*′ and *G*″, (i) logarithmic TTS plot of tan *δ*, and (j) logarithmic plot of *G*′ vs. *G*″. The inset of Figure 4 (h) is the *a*_T_ used (*T*_r_ = 150 °C) and that of the Figure 4 (j) is its enlarged figure, in which the values of *G*′ in terminal region were arbitrarily shifted in the vertical direction. The size of AFM images is 2 × 2 μm^2^.

In comparison to the cases of parallel cylinder-forming SIS + PS2k and SIS + PS4k, perpendicular cylinders were spontaneously produced in SIS + PS12k (*r* = 1.2) at the annealing temperature of 130 °C since the blended PS12k notably enhanced the incompatibility between the block components by virtue of strong affinity between the PS12k and its corresponding PS block [10,11]. The AFM images of Figure 4, which were obtained from ~ 10 *μ*m-thick samples annealed on the Teflon substrates, show the phase behaviors of perpendicular cylinder-forming SIS + PS12k at different temperatures. In the temperature range from 130 °C to 210 °C the gradual orientation transition from perpendicular to parallel was identified with increasing the temperature. The overall perpendicular cylinder orientation at 130 °C [Figure 4 (a)] was thoroughly transferred to parallel cylinder orientation at 210 °C [Figure 4 (e)], via the orientation transition process starting at ~ 150 °C. After achieving complete parallel cylinders, composition fluctuations of block components lasted until ~ 230 °C, and homogeneous melt states were then observed at ~ 240 °C. We recall the fact that such long-lived composition fluctuations detected in perpendicular cylinder-forming SIS + PS12k were not observed in the parallel cylinder-forming SIS + PS2k and SIS + PS4k. Thus, we realize that the stability of transient phases arising from the composition fluctuations at the temperatures above the ODT is largely dependent on the strength of segregation force between the block domains. If the segregation force is not sufficiently strong as in the cases of SIS + PS2k and SIS + PS4k, the composition fluctuations will not be observed in the time scales of stepwise AFM and dynamic mechanical measurements. From the temperature-dependent dynamic mechanical properties, we can also identify the phase behaviors of SIS + PS12k similar to those observed with AFM measurements. In comparison to the cases of parallel cylinder-forming SIS + PS2k and SIS + PS4k, extremely spanned rubbery plateau-like regions were observed for SIS + PS12k over wide temperature and angular frequency ranges, as shown in Figures 4(b), (c), and (d). We attribute this behavior to cylinder orientation which is perpendicular to the shear fields applied and also to strong segregation forces between the block domains. It is notable that the perpendicular cylinder-forming block copolymer mixtures provide more resistance to the externally applied shear force as their PS cylinders are dispersed in a flexural configuration with respect to the shear fields, while in the parallel cylinder-forming block copolymers, the parallel PS cylinders may be more easily aligned in the direction of the applied shear force. In the logarithmic plots of time-temperature superposed *G*′ vs. frequency shown in Figure 4(g), the failure of TTS started to occur at 210 °C, where the orientation transition from perpendicular to parallel terminated (i.e., the ODT temperature ~ 210 °C). After the failure of TTS, the slopes of the plots decreased more steadily to 2 in comparison to the cases of SIS+PS2k and SIS+PS4k, due to the relatively long-term presence of composition fluctuation-driven phases. However, the process of cylinder orientation transition from perpendicular to parallel was not observed in the logarithmic plots of time-temperature superposed *G*′ vs. frequency, even though the amount of parallel cylinders continuously increased with temperature increasing from 150 °C to 210 °C, as shown in the AFM images of Figure 4. Interestingly, however, the orientation transition process was identified in both the low frequency region of time-temperature superposed tan *δ* vs. frequency plots [Figure 4(i)] and the terminal region of the logarithmic plots of *G*′ vs. *G*″ [Figure 4 (j)], which simultaneously contained information on *G*″ as well as *G*′. In the terminal region of *G*′ vs. *G*″ plots [see the inset of the Figure 4(j)], abnormal sudden increases of slopes were found in the temperature range from 150 °C to 170 °C and then extraordinary negativities of slopes were observed at the temperatures ranging from 180 °C to 200 °C, without notable superposition failures in both regions. The former phenomenon suggests abrupt increases of energy dissipation due to the short-ranged cylinder orientation transition from perpendicular to parallel, and the latter, originating from the increase of *G*″ during the decrease of *G*′ [as seen in the terminal regions of Figures 4(d) and (e)], represents more severe energy dissipations due to the relatively longer-ranged orientation transition. After the orientation transition (i.e., above 210 °C), notable thermorheological complexities (i.e., superposition failures) were observed simultaneously with negative slopes in the terminal regions of *G*′ vs. *G*″ plots, and they continued up to ~ 230 °C due to the strong segregation forces between the block components in SIS+PS12k. Such abnormal increases of *G*″ were also found in the low frequency region of logarithmic time-temperature superposed tan *δ* vs. frequency plots above 180 °C [Figure 4 (i)]. In the terminal region, slight failures were observed for TTS corresponding to long-ranged orientation transition at temperatures ranging from 180 °C to 200 °C. Further failures of TTS, representing the termination of perpendicular-to-parallel cylinder orientation transition, started to occur at 210 °C. We note that the tan *δ* vs. frequency plots are less sensitive to the short-range cylinder orientation transition in comparison to the *G*′ vs. *G*″ plots because the slopes of the *G*′ vs. *G*″ plots correspond to the first derivatives of tan^−1^*δ*, which allows the *G*′ vs. *G*″ plots to further illustrate shorter-range (i.e., higher order) phase transitions. Both the logarithmic *G*′ vs. *G*″ plots and the time-temperature superposed tan *δ* vs. frequency plots additionally exhibited relatively shorter-ranged phase transition processes in comparison to the logarithmic plots of the time-temperature superposed *G*′ vs. frequency. We, therefore, conclude that, especially near the terminal region, the *G*′ vs. *G*″ and tan *δ* vs. frequency plots that simultaneously contain the information on *G*′ and *G*″, provide more diverse information on the temperature-dependent phase behaviors of a block copolymer, in comparison to the cases of the *G*′ vs. frequency plots just sensitive to the composition-fluctuation driven global phase transitions.

Based on the results of AFM and dynamic mechanical measurements, we can divide the temperature-dependent microphases of perpendicular cylinder-containing SIS + PS12k with a strong segregation force between the block domains into the following five distinctive microphases and transitions: 1) Perpendicularly oriented cylinder microphases in the temperature range from 130 °C to 150 °C; 2) Perpendicular-to-parallel cylinder orientation transition between 150 °C and 210 °C (the majority of cylinders were oriented perpendicular to the surface at the temperatures ranging from 150 °C to 170 °C, while the majority of cylinders were oriented parallel to the surface in the temperature range from 180 °C to 210 °C); 3) Termination of cylinder orientation transition and the ODT at ~ 210 °C; 4) Long-ranged thermal composition fluctuations from 210 °C to 240 °C due to the strong segregation forces between the block components; and 5) Homogeneous melt states above ~ 240 °C.

In case of perpendicular cylinder-forming SIS + PS12k with a strong incompatibility between the block domains, rheologically measurable long-range orientation transition from perpendicular to parallel was observed before the ODT, and there were long-lived composition fluctuations above the ODT temperature due to the enhanced segregation forces between the block domains. We note that the shift factors used for the TTS of *G*′ were identically applied to the TTS of *G*″, as shown in Figure 4(h). The identical application of shift factors implies that the perpendicular-to-parallel cylinder orientation transition of SIS + PS12k, which occurs in the temperature range between ~ 150 °C and ~ 210 °C, should mainly affect the dynamic mechanical data of *G*″ in the terminal region (i.e., the rheological simplicity of SIS + PS12k is still maintained in the high frequency region below the ODT temperature), and as a consequence, we can use the TTS of *G*′ to predict the ODT temperature of SIS + PS12k and the TTS of *G*″ to estimate its shorter-range phase transitions. It is also notable that such close correlation between dynamic mechanical and AFM measurements, which simultaneously predict the short- and long-range perpendicular-to-parallel cylinder orientation transitions of SIS + PS12k at the similar temperature ranges, strongly supports the fact that the surface morphology observed by AFM measurement does not represent a local cylinder orientation confined only at the sample-superstrate or sample-substrate interfaces, but overall orientation dominant on the whole sample thickness and area.

### 3.4. Phase Behaviors of Perpendicular Cylinder-Forming Block Copolymer Mixture with an Exceptionally Strong Segregation Force between the Block Domains

We note that the perpendicular cylinder-forming SIS + PS15k (*r* = 1.1) will possess stronger affinity between the homologous PS components (i.e., incompatibility between the PS and PI block domains) in comparison to the SIS + PS12k, due to the molecular weight of PS15k being further similar to that of PS block in neat SIS [10,11]. Such greatly enhanced domain-domain incompatibility was morphologically revealed as a perpendicular cylinder orientation of block copolymer cylinders in SIS + PS15k, and the resulting perpendicular orientation did not significantly depend on the external sample fabrication conditions such as the surface energy of substrate, the thickness of sample, and the annealing temperature.

**Figure 5.**
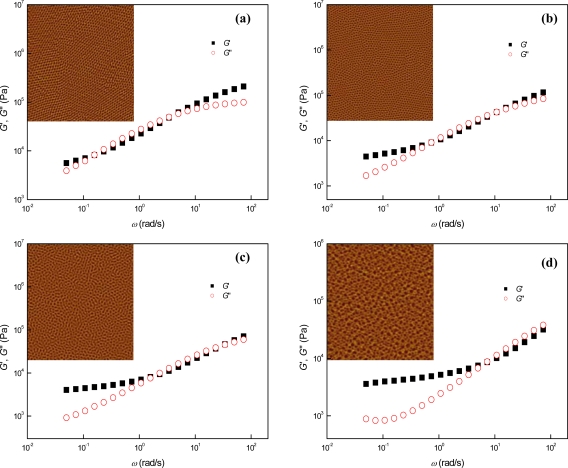

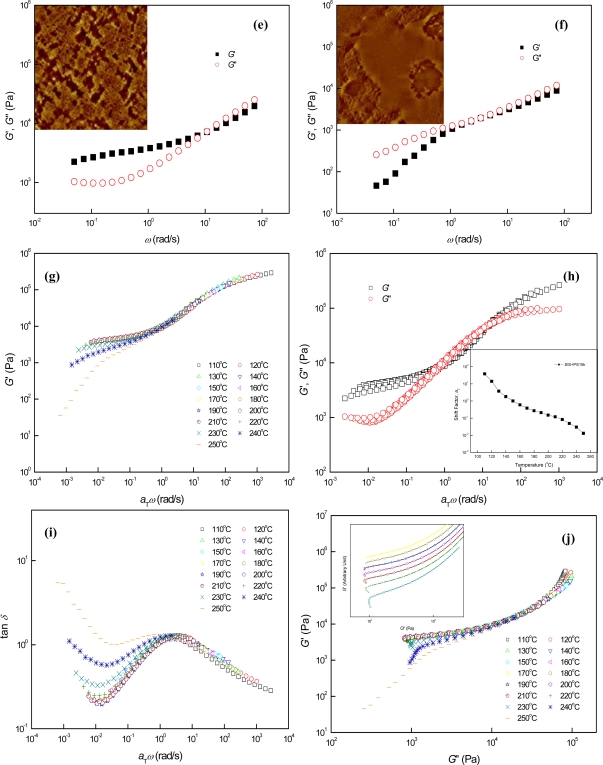
AFM images and dynamic mechanical properties of SIS + PS15k at different temperatures: (a) SIS + PS15k, (b) SIS + PS15k150, (c) SIS + PS15k170, (d) SIS + PS15k210, (e) SIS + PS15k230, (f) SIS + PS15k250, (g) logarithmic TTS plot of *G*′, (h) logarithmic TTS plots of *G*′ and *G*″, (i) logarithmic plot of tan *δ*, and (j) logarithmic plot of *G*″ vs. *G*″. The inset of Figure 5(h) is the *a*_T_ used (*T*_r_ = 150 °C) and that of the Figure 5(j) is its enlarged figure, in which the values of *G*′ in terminal region were arbitrarily shifted in the vertical direction. The size of AFM images is 2 × 2 μm^2^.

The AFM images of Figure 5, which were obtained from ~ 10 *μ*m-thick samples annealed on the Teflon substrates, show the temperature-dependent phase behaviors of SIS+PS15k with an extremely strong segregation force between the block domains. Stable perpendicular cylinders were observed at temperatures ranging from 130 °C to 150 °C as shown in Figures 5(a) and (b), and then hexagonal lattice disordering of perpendicular cylinders was identified in the temperature range between 170 °C and ~ 210 °C [Figures 5(c) and (d)]. During the hexagonal lattice disordering process, the perpendicular cylinders in SIS + PS15k were simply merged to mutually decrease the interfacial area between the PS and PI domains without a perpendicular-to-parallel cylinder orientation transition since the temperature-driven mixing effects did not overwhelm the preference for perpendicular cylinder orientation of SIS + PS15k, which was greatly reinforced by the strong intermolecular affinity between the homologous PS pair. After the termination of hexagonal lattice disordering process, composition fluctuation-driven phases lasted until ~ 230 °C, and homogeneous melt states were then observed at ~ 250 °C.

Dynamic mechanical measurements provided further information on temperature-dependent phase behaviors. Extremely spanned rubbery plateau-like regions were observed in SIS + PS15k over wide temperature and frequency ranges, owing both to the perpendicular cylinder orientation, as well as the strong segregation forces between the block components [Figures 5(c) and (d)]. According to the logarithmic plots of time-temperature superposed *G*′ vs. frequency [Figure 5(g)], the ODT temperature, where the composition fluctuation-driven phases started to occur (i.e., where the hexagonal lattice disordering came to an end), was ~220 °C. Composition fluctuations were observed subsequently until the melt phases were formed at 250 °C. The hexagonal lattice disordering of perpendicular cylinders and their merging processes were observed in the temperature-reduced tan *δ* vs. frequency plots [Figure 5(i)] and the terminal regions of the logarithmic *G*′ vs. *G*″ plots [Figure 5(j)], owing to the abrupt increases of energy dissipation during the transition processes. In the terminal region, the short-ranged hexagonal lattice disordering was observed as sudden increases of slopes in the *G*′ vs. *G*″ plots at temperatures ranging from ~ 170 °C to ~ 190 °C and the long-ranged disordering was detected as both negative slopes in the *G*′ vs. *G*″ plots, as well as slight failures of TTS in the tan *δ* vs. frequency plots at temperatures between ~ 200 °C and ~ 220 °C. Additionally, composition fluctuations were commonly identified in these two plots above ~ 220 °C, both in the form of superposition failures followed by the negativities of slopes in the *G*′ vs. *G*″ plots, and further failures of TTS in the tan *δ* vs. frequency plots.

Judging from the AFM and dynamic mechanical measurements, we can divide the temperature-dependent microphases of perpendicular cylinder-forming SIS + PS15k with an exceptionally strong actual incompatibility between the block domains, into the following five distinctive microphases and transitions: 1) Perpendicularly oriented cylinder microphases in the temperature range from 130 °C to 160 °C; 2) Hexagonal lattice disordering of perpendicular cylinders between 170 °C and 220 °C (the short-ranged hexagonal lattice disordering at the temperatures ranging from 170 °C to 190 °C, followed by the long-range hexagonal lattice disordering in the temperature range from 200 °C to 220 °C); 3) Termination of the lattice disordering and the ODT at ~ 220 °C; 4) Long-ranged thermal composition fluctuations from 220 °C to 250 °C due to the strong segregation forces between the block components; and 5) Homogeneous melts above ~ 250 °C.

In case of a perpendicular cylinder-forming block copolymer material with a notably increased incompatibility between the block components, rheologically detectable long-range hexagonal lattice disordering was identified before the ODT, and there were also long-lived composition fluctuations above the ODT temperature. We note that the shift factors used for the TTS of *G*′ were identically applied to the TTS of *G*″, as shown in Figure 5(h). The identical application of shift factors implies that the hexagonal lattice disordering process of SIS+PS15k, which occurs in the temperature range between ~170 °C and ~220 °C, should mainly affect the dynamic mechanical data of *G*″ in the terminal region (i.e., the rheologically simple behavior of SIS + PS15k is still maintained in the high frequency region below the ODT temperature), and consequently, we can use the TTS of *G*′ to predict the ODT temperature of SIS + PS15k and the TTS of *G*″ to estimate its shorter-range phase transitions.

## 4. Conclusions

In summary, we have categorized three different temperature-dependent phase behaviors of miscible cylinder-forming block copolymers in accordance with the strength of segregation forces between the block domains: 1) In case of a parallel cylinder-forming block copolymer with a relatively weak segregation force between the block domains, arising from weak intermolecular affinity between the homologous polymer pair (for example, SIS + PS2k and SIS + PS4k), parallel cylinder-straightening process was observed before the ODT, and there were no long-range composition fluctuations near the ODT temperature; 2) In case of a perpendicular cylinder-forming block copolymer with a strong segregation force between the block domains, arising from relatively strong intermolecular affinity between the homologous PS pair (for example, SIS + PS12k), rheologically measurable long-range orientation transition from perpendicular to parallel was observed before the ODT, and there were long-lived composition fluctuations above the ODT temperature; and 3) In case of a perpendicular cylinder-forming block copolymer with an exceptionally strong segregation force between the block domains, arising from much stronger intermolecular affinity between the homologous polymer pair, rheologically detectable long-range hexagonal lattice disordering was identified before the ODT, without a perpendicular-to-parallel cylinder orientation transition, and there were also long-lived composition fluctuations above the ODT temperature. Therefore, we conclude that the amount of segregation forces between the block domains is a critical factor to determine the temperature-dependent phase behaviors of a block copolymer, including block copolymer domain orientation below the ODT temperature, as well as the temperature range of composition fluctuation above the ODT temperature. From a viewpoint of nanotechnological applications of block copolymers, our illustrated guidelines on block copolymer phase behaviors should provide practical and precise information required for the fabrication of well-defined block copolymer nanostructures.

## Figures and Tables

**Table 1.** Thermal histories of block copolymer samples used.

**Sample**	**Thermal History**
SIS, SIS + PS2k, SIS + PS4k, SIS + PS12k, and SIS + PS15k	Annealed at 130 °C for 50 h and then rapidly quenched in liquid nitrogen (LN).
SIS150, SIS + PS2k150, SIS + PS12k150, and SIS + PS15k150	First annealed at 130 °C for 47.5 h and then heated to 150 °C and held there for 2.5 h and then rapidly quenched in LN.
SIS170, SIS + PS4k170, SIS + PS12k170 and SIS + PS15k170	First annealed at 130 °C for 47.5 h and then heated to 150 °C and held there for 1 h and then heated to 170 °C and held there for 1.5 h and then rapidly quenched in LN.
SIS + PS4k190 and SIS + PS12k190	First annealed at 130 °C for 47.5 h and then heated to 150 °C and held there for 1 h and then heated to 170 °C and held there for 1 h and then heated to 190 °C and held there for 0.5 h and then rapidly quenched in LN.
SIS-210, SIS+PS4k210, SIS+PS12k210 and SIS+PS15k210	First annealed at 130 °C for 47.5 h and then heated to 150 °C and held there for 1 h and then heated to 170 °C and held there for 0.5 h and then heated to 190 °C and held there for 0.5 h and then heated to 210 °C and held there for 0.5 h and then rapidly quenched in LN.
SIS + PS12k230 and SIS + PS15k230	First annealed at 130 °C for 47.5 h and then heated to 150 °C and held there for 0.5 h and then heated to 170 °C and held there for 0.5 h and then heated to 190 °C and held there for 0.5 h and then heated to 210 °C and held there for 0.5 h and then heated to 230 °C and held there for 0.5 h and then rapidly quenched in LN.
SIS + PS15k250	First annealed at 130 °C for 47.5 h and then heated to 150 °C and held there for 0.5 h and then heated to 170 °C and held there for 0.5 h and then heated to 190 °C and held there for 0.5 h and then heated to 210 °C and held there for 0.5 h and then heated to 230 °C and held there for 0.5 h and then heated to 250 °C and held there for 0.5 h rapidly quenched in LN.
